# The impact of physical activities on adolescents’ rule consciousness: the chain mediation effect of friendship quality and emotional intelligence

**DOI:** 10.3389/fpubh.2025.1581016

**Published:** 2025-06-02

**Authors:** Jiahui Peng, Li Cao, Deqiao Zhou

**Affiliations:** ^1^School of Physical Education and Sport Science, Qufu Normal University, Jining, China; ^2^School of Physical Education¸Shandong Normal University, Jinan, China; ^3^School of Physical Education, Shandong University, Jinan, China

**Keywords:** physical activity, teenagers, rule consciousness, friendship quality, emotional intelligence

## Abstract

**Introduction:**

With the increasing emphasis on the development of adolescents’ character and behavior in modern society, how to effectively cultivate adolescents’ rule awareness has become a pressing issue in the field of education. Despite the increasing emphasis on developing adolescents’ character and behavior, educators face significant challenges in cultivating rule awareness. Traditional educational practices often fall short in integrating physical activity with socio-emotional learning, and they struggle to capture the complex dynamics influencing rule adherence. Rule awareness is not only a crucial foundation for social behavioral norms but also has a profound impact on adolescents’ personal growth and social adaptability. This study investigates the effect of physical activity on adolescents’ rule awareness and reveals the chain mediation effects of friendship quality and emotional intelligence.

**Methods:**

To explore the relationship between physical activity and adolescents’ rule awareness and analyze the mediating and chain mediating roles of friendship quality and emotional intelligence, a questionnaire survey was conducted with 612 adolescents aged 8–16. Data were analyzed using SPSS 27.0 and PROCESS 4.0 for correlation analysis, regression analysis, and mediation effect testing.

**Results:**

Physical activity significantly and positively predicted adolescents’ rule awareness. Friendship quality and emotional intelligence played independent mediating roles and formed a chain mediation effect in this process. The study’s findings reveal the complex mechanisms through which physical activity, friendship quality, and emotional intelligence influence adolescents’ rule awareness, providing theoretical foundations and practical insights for further investigating important factors affecting adolescents’ rule awareness, effectively curbing rule violations, and continuously strengthening rule awareness development.

**Conclusion:**

To further enhance adolescents’ rule awareness and maintain social order and stability, both schools and society should create a favorable sporting environment and reasonably increase the amount of physical activity among adolescents.

## 1 Introduction

The conceptualization of rule consciousness in academic discourse exhibits inherent multidimensionality, with adolescents’ rule consciousness remaining ambiguously defined. Contemporary sociological perspectives posit that rule consciousness encompasses not merely rational cognition of institutional norms, but more crucially, the transformative process through which external constraints are internalized into self-regulatory behaviors—a critical indicator of individual socialization (1). Institutional economics emphasizes its social functionality, identifying rule consciousness as the cornerstone for effective institutional operations that reduce enforcement costs and facilitate social cooperation (2). Ethicists further delineate affective, rational, and spiritual dimensions of rules, asserting that benign regulations must align with humanitarian principles, civil rights protection, and openness (3). Synthesizing prior conceptualizations, this study operationalizes adolescent rule consciousness as: a dynamic cognitive system formed through internalized comprehension of external norms, functional understanding of social contracts, and critical integration of ethical values during socialization. Its essence lies in the transformative mechanism from passive compliance to active construction, simultaneously driving individual self-discipline and sustaining intergenerational continuity of social collaboration systems.

Despite sustained policy and educational interventions, deficient rule consciousness persists as a salient developmental issue among adolescents. Prevalent phenomena such as school bullying (4) and cyber misconduct (5) reflect systematic neglect of social norms within certain youth cohorts. This trend intensifies amidst the dual pressures of value pluralism during social transition and the eroded boundaries of virtual space regulations in the digital era (6). Consequently, investigating effective mechanisms for rule internalization bears urgent practical significance. Physical activities warrant particular attention given their inherent capacity for peer interaction and emotional regulation.

**Figure 1:**
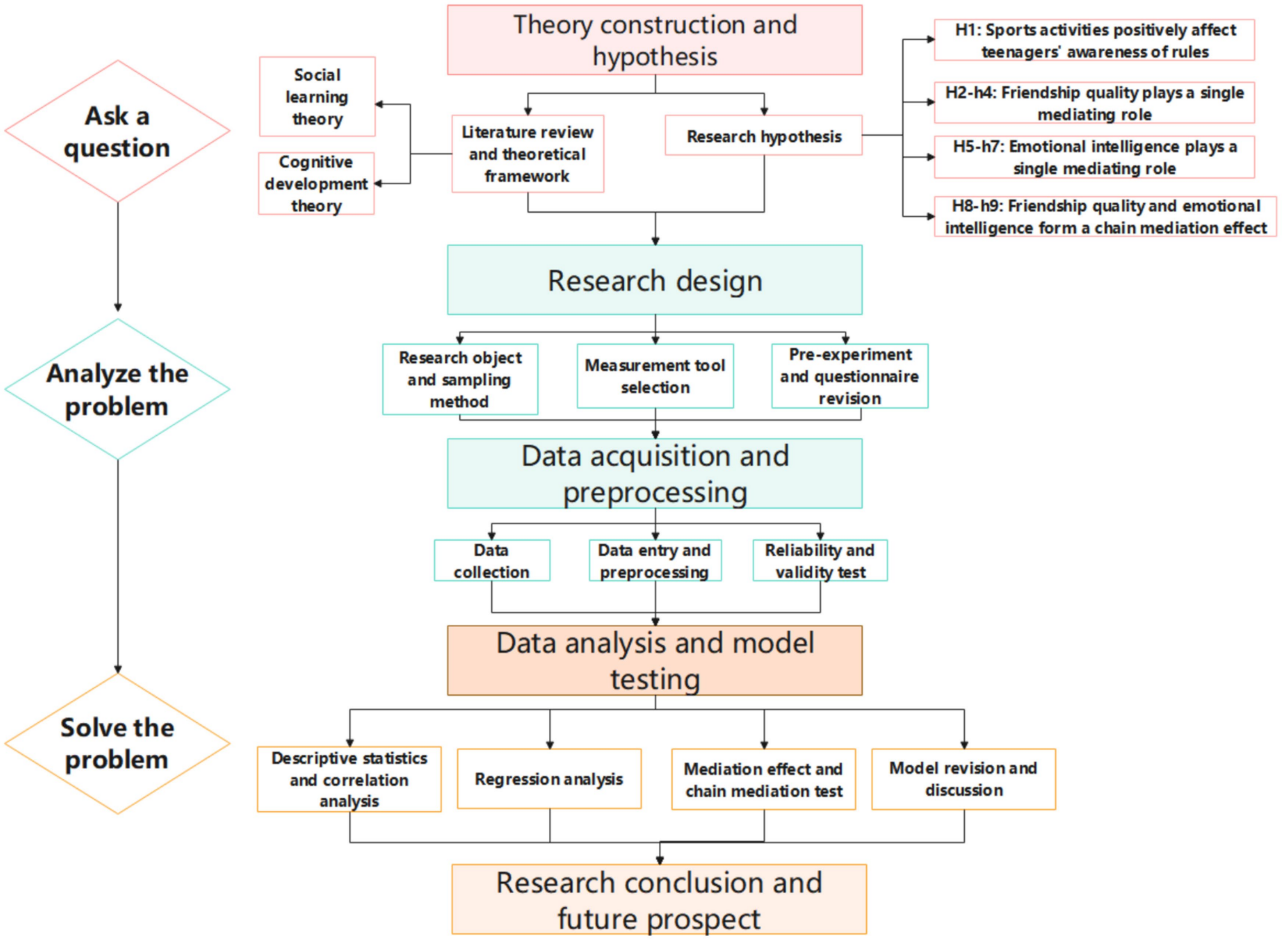
Technical route.

As concrete practice fields for rule embodiment, structured sports provide adolescents with explicit cognitive frameworks through codified athletic regulations, demonstrating unique advantages in cultivating rule consciousness (7). Within standardized sporting environments, participants not only adhere to explicit rules but progressively internalize normative behaviors through sustained team interactions and competitive engagements. Empirical evidence confirms that athletic participation enhances students’ rule awareness, compliance efficacy, and practical application, fostering norm-governed behaviors including mutual respect, proactive cooperation, and equitable competition (8). The quintessential sports ethos—respect, adherence, compliance, and fair play—provides theoretical substantiation for this cultivation mechanism (9). Furthermore, as microcosms of social order, sports activities enhance individuals’ grasp of procedural justice while strengthening collective rule internalization through team-based coordination (10). This study systematically investigates how physical activities serve as an effective vehicle for cultivating adolescents’ rule consciousness (see Figure 1).

## 2 Literature review and research hypothesis

### 2.1 Sports activities and adolescent rule consciousness

Previous scholarship has substantiated the significance of sports participation in enhancing adolescent rule consciousness through both theoretical and empirical lenses. Theoretically, Bandura’s (11) social learning theory posits that individuals acquire social norms and behavioral codes through observing and emulating role models. Given the inherent collectivity and rule-bound nature of sports, adolescents gain systematic opportunities to observe and replicate normatively appropriate behaviors. This mechanism suggests a positive correlation between sports engagement and increased adherence to social norms and prosocial conduct.

Empirical investigations further validate the rule-enhancing effects of athletic involvement. Weiss et al. (12) conducted a longitudinal study of U.S. adolescents, revealing that team sports participation correlated with heightened rule compliance and strengthened fair play awareness. Holt and colleagues (13) demonstrated that discipline and teamwork cultivated through sports participation transcended athletic contexts, generating positive behavioral spillovers in familial and educational environments. Advanced analyses indicate that students with frequent sports participation significantly outperform their less-active peers in both depth of rule comprehension and consistency of norm enforcement (14). From a neurocognitive perspective, sustained athletic training enhances prefrontal cortex functionality in impulse inhibition, with such neuroplastic changes providing physiological substrates for rule consciousness development (15).

Collectively, theoretical frameworks and systematic empirical evidence converge to demonstrate the substantive role of physical activities in cultivating adolescent rule consciousness. By integrating social learning theory with insights from systematic literature review, the present discussion establishes a robust foundation for hypothesizing that organized sports participation facilitates social norm internalization. Accordingly, this study proposes the following hypothesis:

H1: Sports activities positively influence adolescents’ rule consciousness.

### 2.2 The mediating role of friendship quality

This study constructs an analytical framework by interweaving Bronfenbrenner’s ecological systems theory, which delineates hierarchical influence mechanisms, with the stage-specific characteristics of adolescents’ socioemotional development. This dual theoretical grounding clarifies the rationale for selecting mediating variables.

From a developmental psychology perspective, adolescence marks a critical transition from vertical family relationships to horizontal peer relationships, during which friendship quality exhibits time-sensitive predictive power over psychosocial adaptation (16). Concurrently, the ecological systems perspective reveals that proximal interpersonal processes—such as friendship quality—provide more direct explanations for individual psychological mechanisms compared to macro-level variables like school climate (17). By integrating developmental chronology with systemic hierarchies, this study establishes the theoretical appropriateness of friendship quality as the core mediator.

The principal mechanism through which physical activities shape rule consciousness operates via three dimensions of friendship quality. (1) Interactive Reinforcement Dimension: Team-based social interactions create co-constructed spaces for rule cognition. Through role allocation and tactical negotiations in sports contexts, adolescents establish shared rule-understanding frameworks within peer relationships (18). (2) Emotional Scaffolding Dimension: Peer empathy during athletic setbacks facilitates rule internalization. Empirical evidence indicates that emotional security derived from high-quality friendships strengthens adolescents’ value alignment with competitive norms (19). (3) Social Learning Dimension: Observational learning within peer networks promotes behavioral internalization of rules. As emphasized by social learning theory, the mechanisms of modeling and imitation in behavioral acquisition remain equally operative in athletic settings (11).

These interconnected pathways collectively form a comprehensive “cognitive-affective-behavioral” chain of influence. Based on this theoretical synthesis, we postulate the following hypotheses:

H2: Physical activity can positively affect peer friendship

H3: Peer friendship positively influences adolescents' rule consciousness.

H4: Peer friendship mediates the relationship between sports activities and adolescents' rule consciousness.

### 2.3 The mediating role of emotional intelligence

This study selects emotional intelligence (EI) as the central mediating variable, grounded in Social Cognitive Theory and the Emotion-Cognition Integration Model, for three theoretical reasons. First, as a core competency for social adaptation, EI elucidates the pathway through which physical activity influences cognitive construction via affective experiences. Empirical evidence confirms that emotion regulation training in sports contexts significantly enhances adolescents’ emotional awareness and regulatory capacities (20). Second, EI and rule awareness exhibit a bidirectional constructive relationship. Individuals with higher EI demonstrate superior comprehension of implicit social interaction norms (21), while rule internalization inherently requires emotional regulation skills (22). Third, compared to alternative mediators (e.g., self-control, moral reasoning), EI demonstrates stronger contextual transferability—bridging immediate emotional experiences during physical activity with sustained adherence to societal rules in daily life.

Emotional intelligence serves as a bridge between sports activities and rule consciousness. The rules in sports are not merely external constraints, but also internal behavioral guidelines. By learning how to follow rules in sports activities, adolescents are also learning how to manage their emotions and behavior. Studies have shown that athletes with stronger Emotional intelligence abilities are better able to understand and comply with competition rules, and exhibit higher levels of self-discipline during games (23). Emotional intelligence is closely linked to adolescents’ social adaptability. Emotional intelligence mediates the relationship between sports activities and adolescents’ implicit behavioral issues, highlighting its significance in adolescent mental health (24). This finding further supports the mediating role of Emotional intelligence between sports activities and rule consciousness.

Moreover, Emotional intelligence can also indirectly influence rule consciousness by impacting adolescents’ social cognition. Social cognition refers to the ability to understand and interpret others’ behaviors during social interactions, and it is closely related to Emotional intelligence. Adolescents with stronger Emotional intelligence skills are better at understanding others’ emotions and intentions, which enables them to better follow social rules (25). In sports activities, this social cognition is especially important, as sports often require individuals to cooperate with others in a team setting, and this cooperation is fundamentally based on understanding and adhering to rules. Based on these findings, we propose the following hypotheses:

H5: Physical activity can positively affect emotional intelligence

H6: Emotional intelligence positively influences adolescents' rule consciousness.

H7: Emotional intelligence mediates the relationship between sports activities and adolescents' rule consciousness.

### 2.4 The chain mediating role of friendship quality and emotional intelligence

Extant research establishes physical activities as a pivotal avenue for fostering adolescents’ social development, with mechanisms including team collaboration, competitive engagement, and rule-based practice exerting positive impacts on rule consciousness (26). Concurrently, the collective participation environments cultivated through sports activities amplify the formation and maintenance of peer friendships. As a core component of social support networks, peer friendships not only facilitate cognitive internalization of group norms via social learning mechanisms (27) but may also enhance emotional regulation capacity through affective reciprocity and role modeling (28).

Emotional management capacity, serving as a critical psychological resource for adapting to societal rules, proves particularly vital in competitive athletic contexts. Individuals with heightened emotional intelligence demonstrate greater propensity for conflict resolution through rational modalities, thereby deepening their comprehension of rule necessity (29). Notably, the quality of peer friendships may indirectly influence adolescents’ emotion-regulation strategies under stressful conditions—through provisions of emotional security and behavioral feedback—which subsequently modulates their rule acceptance (30). This dynamic interplay suggests that sports participation may function as a social laboratory for practicing emotion-regulation skills via the facilitation of peer relational networks, ultimately establishing a sequential developmental pathway: sports engagement → social bonding → emotional self-regulation → rule internalization (31). Building upon these conceptual linkages, this study advances the following hypotheses:

H8: Friendship quality can positively affect emotional intelligence

H9: Friendship quality and emotional intelligence may serve as chain mediators in the relationship between sports activities and adolescents' rule consciousness.

## 3 Research methods

### 3.1 Research subjects

The research subjects in this study are adolescents aged 8 to 16, focusing on the impact of sports activities on rule consciousness and the chain mediating roles of emotional intelligence and friendship quality. A sample size estimation was conducted using G*Power 3.1 software, with an alpha level of 0.05, a statistical power (1-*β*) of 0.99, and a medium effect size (0.15). The result indicated that a sample size of 203 would be sufficient.

This study adopted a stratified random sampling approach by dividing Shandong Province into three regions based on spatial geography: eastern, central, and western. Specifically, the study selected Qingdao (Eastern Shandong), Jinan (Central Shandong), and Jining (Western Shandong) as survey cities. Within each city, one urban public primary school, one urban public junior high school, one township public primary school, and one township public junior high school were randomly chosen as survey sites. This sampling strategy ensures a representative coverage of different educational settings (urban vs. township) as well as regional differences in economic and cultural backgrounds across Shandong Province.

Participants were adolescents aged 8–16 years. Although the World Health Organization typically defines adolescents as those aged 10–19 years, the chosen age range is justified by two considerations. First, in China, children aged 8 (generally in third grade or above) have developed sufficient reading comprehension skills to accurately complete the questionnaires. Second, 16-year-old students, who are usually in junior high school (below ninth grade), are predominantly day students with relatively lower academic pressures and more stable availability for survey participation. Furthermore, considering that early childhood social cognition may have implications for later adolescent behavior, the age range was slightly broadened to include younger participants. It is acknowledged that individual differences in age, gender, and other factors inevitably affect social cognitive levels; therefore, these variables are controlled in subsequent analyses to minimize their impact on the research results.

Questionnaires were administered collectively within administrative classes. For younger participants, homeroom or Chinese language teachers assisted in explaining survey items to ensure comprehension. A total of 650 paper questionnaires were distributed. After excluding 38 invalid responses (due to patterned or incomplete answers), 612 valid questionnaires were retained, yielding a 94.3% valid response rate. The sampling details are summarized in Table 1, confirming the sample size meets statistical standards.

**Table 1:** Distribution of demographic variables of respondents.

Variable	Class	Frequency	Percentage
Age	8 ~ 10	149	24.3%
	11 ~ 13	269	44%
	14 ~ 16	194	31.7%
Sex	Male	339	55.4%
	Female	273	44.6%
Place of residence	Village	143	23.4%
	City	469	76.6%
	Total	612	100%

### 3.2 Survey tools

#### 3.2.1 Sports activity level scale

Physical activity levels were assessed using the Physical Activity Rating Scale-3 (PARS-3), revised by Liang (32). This scale evaluates exercise volume through three dimensions: intensity, frequency, and duration, rated on a 5-point Likert scale. The total activity score was calculated using the formula: Activity Score = Frequency × (Duration−1) × Intensity, yielding a range from 0 to 100 points. Participants were categorized into three tiers based on their scores: low activity (≤19 points), moderate activity (20–42 points), and high activity (43–100 points).

To enhance response accuracy across age groups, contextual prompts were embedded in subjective items. For example, the intensity dimension included:

Light intensity: e.g., walking, calisthenics.
Low-moderate intensity: e.g., recreational volleyball, table tennis, jogging.
Moderate-vigorous intensity: e.g., cycling, running, table tennis (sustained).
High intensity (non-sustained): e.g., badminton, basketball, tennis, football (characterized by rapid breathing and profuse sweating).
High intensity (sustained): e.g., sprinting, aerobics routines, swimming.

The scale demonstrated strong construct validity in this study, with a Kaiser-Meyer-Olkin (KMO) value of 0.905 and Bartlett’s test of Sphericity (χ^2^ = 5,623.241, *p* < 0.001). Internal consistency was acceptable, as indicated by a Cronbach’s *α* coefficient of 0.70.

#### 3.2.2 Rule consciousness questionnaire

Based on existing mature scales and Kohlberg’s theory of moral development, this study divides adolescents’ rule consciousness into three dimensions: rule understanding, rule compliance, and rule internalization. Additionally, based on social cognitive theory, the questionnaire adds three more dimensions: rule perception, rule application, and rule reflection. It also incorporates the influence of external environments on the development of rule consciousness within Kohlberg’s framework.

Six experts in psychology, education, and sports were invited to use the Delphi method to scientifically assess the dimensions and items of the scale. Subsequently, three elementary school teachers and three primary school students evaluated the readability of the questionnaire, and unclear items were revised and optimized. Based on feedback, similar items were merged, duplicate items were deleted, and items unrelated to this study were removed. The revised questionnaire was then reviewed by experts in a second round of consultation. Results showed that the variation coefficient of all items was less than 0.25, meeting the relevant standards, forming the initial version of the questionnaire.

To ensure the scientific and reasonable design of the questionnaire, a pilot survey was conducted, and cross-validation was used to test the model. In the pilot phase, 80 primary school students from off-campus training classes in Jining City were surveyed, yielding 76 valid responses, with an effective response rate of 95%. SPSS 26.0 software was used to analyze the reliability of the pilot data. The factor loadings of all items were greater than 0.45, and the Cronbach’s *α* coefficient of each item was above 0.6, indicating good reliability, and all items were retained (see Table 2).

**Table 2:** Reliability test results of adolescent rule awareness scale.

Dimension	Question number	Item	Cα
Rule understanding	Q1	I know that I should not talk casually during class.	0.933
	Q2	I understand why it’s important to watch the traffic lights when crossing the road.	0.933
	Q3	I know that games have rules, and classrooms have rules.	0.929
	Q4	I know that rules are meant to keep everyone safe and happy.	0.931
Rule compliance	Q5	When the teacher asks us to line up, I stand in line obediently.	0.930
	Q6	I focus on listening during class and do not engage in other activities.	0.929
	Q7	When playing a game, I follow the rules and do not cheat.	0.931
	Q8	I do not break the rules just because no one is watching.	0.933
Rule internalization	Q9	I believe rules are made for the good of everyone, not for punishing people.	0.929
	Q10	I follow the rules not only because the teacher might reprimand me.	0.933
	Q11	When there are no clear rules, I think about what the right thing to do is.	0.930
	Q12	I believe following the rules helps me get along better with my classmates.	0.931
Rule perception	Q13	I think the school rules are fair.	0.930
	Q14	If I do not understand the rules, I will ask the teacher or my parents.	0.930
	Q15	I believe rules help us get along and cooperate better.	0.930
Rule application	Q16	When I have conflicts with my classmates, I try to resolve them using the rules.	0.931
	Q17	If someone breaks the rules, I remind them.	0.929
	Q18	When I go to a new place, I first try to understand the rules there.	0.930
	Q19	I use rules to help solve problems.	0.931
Rule reflection	Q20	If I feel I have not followed the rules, I reflect on why.	0.930
	Q21	I believe following the rules is to make everyone happy, not to receive praise.	0.932
	Q22	I always think about how I can use the rules better.	0.929

As a result, the final questionnaire of this study consists of six dimensions: rule understanding, rule compliance, rule internalization, rule perception, rule application, and rule reflection, with a total of 22 items. The KMO value of the scale was 0.97, and Bartlett’s Test of Sphericity was 10332.669 (*p* < 0.0001), indicating that the scale has good construct validity. The Cronbach’s *α* coefficient was 0.96, demonstrating excellent reliability.

#### 3.2.3 Friendship quality questionnaire

This study uses a shortened version of the Friendship Quality Questionnaire developed by Parker and Asher in 1993 and adapted by Zhou et al. (33). The scale contains 18 items, which assess six dimensions: companionship and recreation, affirmation and care, intimacy and disclosure, assistance and guidance, conflict resolution, and conflict and betrayal. The first five dimensions reflect positive friendship quality, while the conflict and betrayal factor reflect negative friendship quality. The scale uses a 5-point Likert self-report scoring system. After reversing the scores for the conflict and betrayal dimension, the total score is calculated. A higher total score indicates a higher level of friendship quality. The KMO test value of the scale is 0.95, and Bartlett’s test of Sphericity is 8311.025 (*p* < 0.0001), indicating that the structural validity of the scale is good. The Cronbach’s *α* coefficient of the questionnaire is 0.70, indicating good reliability.

#### 3.2.4 Emotional intelligence scale

This study uses the Emotional Intelligence Scale developed by Schutte et al. and revised by Wang Caikang to measure the emotional intelligence of parents/guardians (34). The scale consists of 33 items, which assess four dimensions: emotional perception, self-regulation of emotions, regulation of others’ emotions, and emotional utilization. The scale uses a 5-point Likert self-report scoring system, with the third item being reverse scored and all other items being positively scored. A higher score indicates higher emotional intelligence. The KMO test value of the scale is 0.92 (*p* > 0.5), and Bartlett’s test of Sphericity is 8216.085 (*p* < 0.0001), indicating that the structural validity of the scale is good. The Cronbach’s *α* coefficient of the questionnaire is 0.89, indicating good reliability.

### 3.3 Data processing and analysis

SPSS 27.0 and PROCESS 4.0 software were used for statistical analysis of the relevant data. The following operations were conducted: (1) Classification, transformation, and calculation of valid data; (2) Harman’s single-factor test was applied to check for common method bias; (3) The Cronbach’s *α* coefficient and Bartlett’s test of Sphericity were analyzed to assess the reliability and structural validity of the measurement tools; (4) Correlation analysis and linear regression were used to verify the correlations between variables and whether physical activity, friendship quality, and emotional intelligence significantly affect adolescent rule awareness; (5) The Bootstrap mediation effect analysis method was employed to test the individual and chain mediation effects of friendship quality and friendship emotional intelligence in the relationship between physical activity and adolescent rule awareness.

## 4 Research results

### 4.1 Common method bias test

To mitigate common method bias, we employed a coded anonymous assessment approach during data collection, effectively controlling potential sources of bias. Additionally, we conducted an exploratory factor analysis (EFA) on all test items using SPSS 27.0 and applied Harman’s single-factor test. The results indicated that the first factor explained 32.60% of the variance, which is below the 40% threshold (35), suggesting that this study does not suffer from significant common method bias.

### 4.2 Correlation analysis of research variables

We performed correlation analyses among the four primary research variables: physical activity, friendship quality, emotional intelligence, and adolescents’ rule awareness (see Table 3). The results revealed significant positive correlations among these variables, indicating that they are interrelated. These findings support hypotheses H1, H2, H3, H4, H5, H6, H7, H8 and H9, and meet the statistical requirements for further mediation effect analysis involving friendship quality and emotional intelligence.

**Table 3:** Means, standard deviations, and correlation coefficients of research variables.

	M	SD	PA	RC	FQ	EI
PA	18.89	16.66	1			
RC	86.63	25.44	0.434**	1		
FQ	42.12	16.43	0.344**	0.560**	1	
EI	110.04	15.63	0.468**	0.578**	0.570**	1

*p < 0.05; **p < 0.01; ***p < 0.001; Physical activity (PA), Rule consciousness (RC), Friendship quality (FQ), Emotional intelligence (EI). The same significance levels apply throughout this manuscript.

Three hierarchical regression analyses were performed to examine the individual predictive effects of physical activity, friendship quality, and emotional intelligence on adolescents’ rule consciousness. Using the forced entry method, with each predictor entered separately into the regression model, the results revealed statistically significant associations (all *p* < 0.001): (1) Physical activity demonstrated a substantial positive influence (*β* = 0.434, *p* < 0.001), explaining 18.7% of the variance in rule consciousness; (2) Friendship quality emerged as a stronger predictor (*β* = 0.560, *p* < 0.001), accounting for 31.2% of the variance; (3) Emotional intelligence showed the strongest predictive power (*β* = 0.578, *p* < 0.001), contributing to 33.3% of the variance in rule consciousness; (4) Age also had a positive effect on rule consciousness (β = 0.266, *p* < 0.001), contributing to 6.9% of the variance in rule consciousness; (5) Urban and rural difference positively affected rule consciousness (β = 0.238, *p* < 0.001), contributing to 5.5% of the variance in rule consciousness; (6) Gender difference had no significant effect on rule awareness (*p* > 0.5) (see Table 4).

**Table 4:** Individual regression analysis of physical activity, friendship quality and emotional intelligence on adolescents’ rule awareness.

Variable	Adolescent rule consciousness						
	B	SE	β	T	F	R^2^	R^2^_adj_
PA	0.663	0.056	0.434	11.913	141.921	0.189	0.187
FQ	1.225	0.073	0.560	16.689	278.514	0.313	0.312
EI	0.941	0.054	0.578	17.512	306.666	0.335	0.333
Age	2.835	0.417	0.266	6.805	46.309	0.071	0.069
Place of Residence	14.278	2.362	0.238	6.045	36.538	0.057	0.055

R^2^_adj_ indicates adjusted R^2^.

### 4.3 Intermediate effect test

Bootstrap Analysis with 5,000 Resamples: Mediating Effects of Friendship Quality and Emotional Intelligence in the Relationship between Physical Activity and Adolescents’ Rule Awareness, and Confidence Intervals (Table 5). The results of the analysis indicated that the total effect of physical activity on adolescents’ rule awareness was 0.625. The 95% confidence intervals (LLCL = 0.519, ULCL = 0.731) for the mediating effects of both friendship quality and emotional intelligence did not include zero, suggesting that physical activity significantly influences adolescents’ rule awareness through these two mediators. The direct effect of physical activity on adolescents’ rule awareness was 0.252 (direct path), and the 95% Bootstrap confidence interval (LLCL = 0.152, ULCL = 0.352) did not include zero, indicating a significant direct effect. This direct effect accounted for 40.3% of the total effect, supporting hypotheses H1.

**Table 5:** Results of intermediate effect path test.

Effect	Path	Effect	Boot SE	LLCL	ULCL	Effect ratio (100%)
Total effect	Direct path	0.625	0.054	0.519	0.731	100
Direct effect		0.252	0.051	0.152	0.352	40.3
Total mediating effect		0.373	0.038	0.304	0.452	59.7
Mediating effect	Ind1	0.146	0.026	0.100	0.201	23.4
	Ind2	0.152	0.025	0.108	0.205	34.3
	Ind3	0.075	0.012	0.053	0.101	12.0

The model reveals that physical activity can predict adolescents’ rule awareness, with friendship quality and emotional intelligence acting as indirect mediators through three distinct pathways. The total indirect effect was 0.373, and the 95% Bootstrap confidence interval (LLCL = 0.038, ULCL = 0.304) did not include zero, accounting for 59.7% of the total effect.

**Figure 2:**
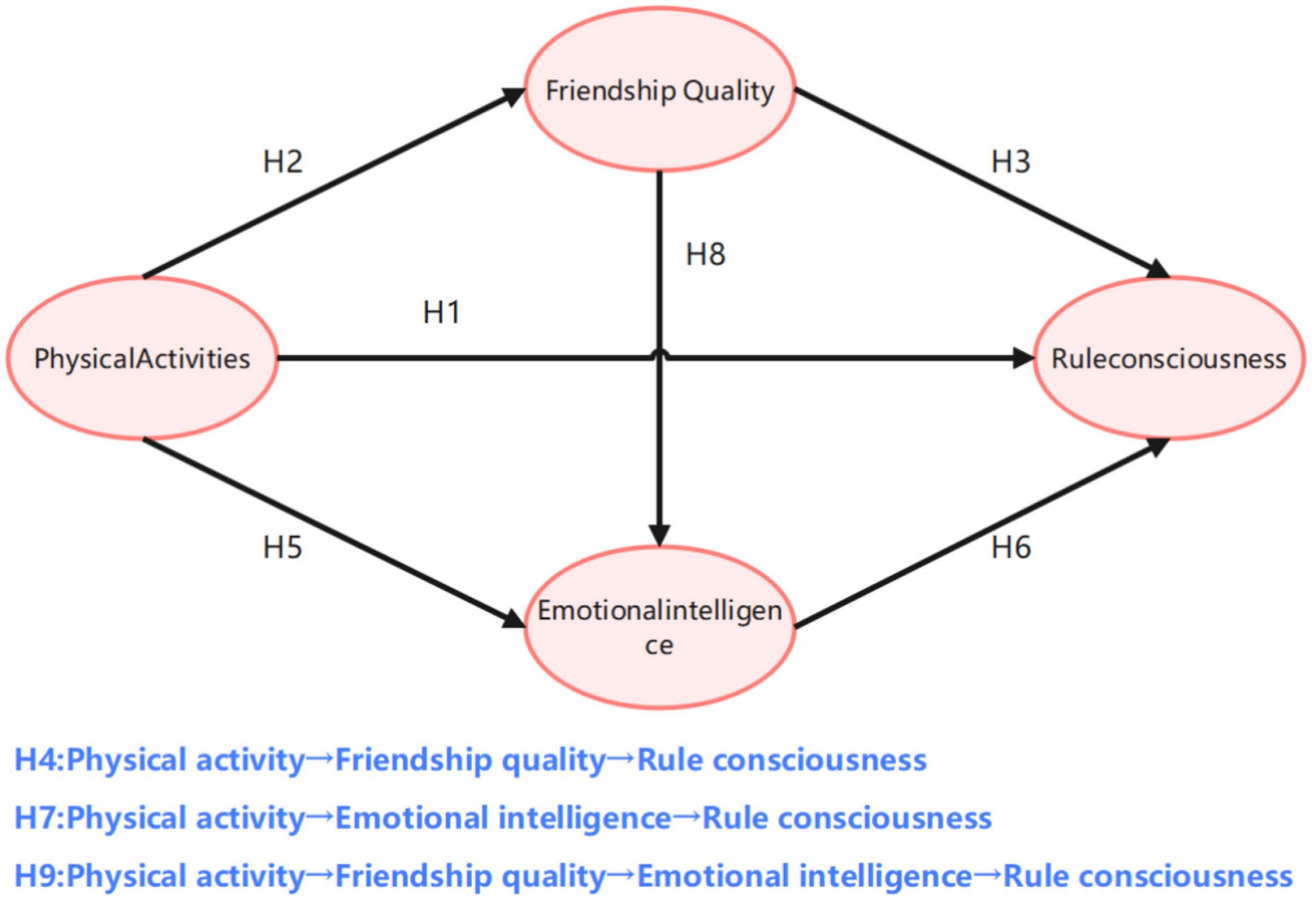
Hypothesis model.

**Figure 3:**
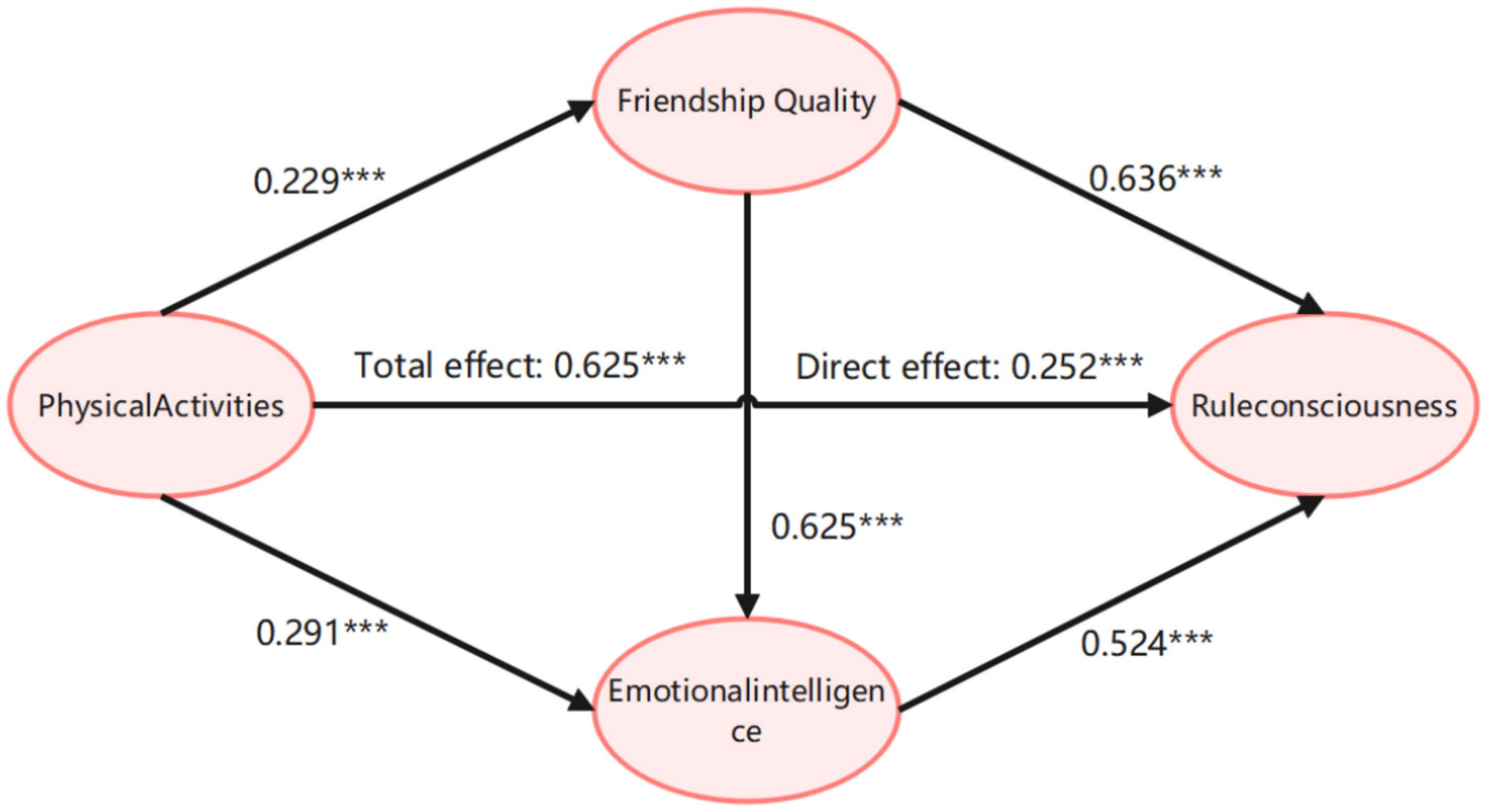
A chain mediation model between friendship quality and emotional intelligence on the effect of physical activity on adolescents’ awareness of rules.

Specifically, the first mediation pathway (Path 1: Physical Activity → Friendship Quality → Adolescents’ Rule Awareness) showed an indirect effect of 0.146, contributing 23.4% of the total effect, supporting hypotheses H2, H3 and H4. The second pathway (Path 2: Physical Activity → Emotional Intelligence → Adolescents’ Rule Awareness) yielded an indirect effect of 0.152, accounting for 34.3% of the total effect, supporting hypotheses H5, H6 and H7. The third chain mediation pathway (Path 3: Physical Activity → Friendship Quality → Emotional Intelligence → Adolescents’ Rule Awareness) produced an indirect effect of 0.075, contributing 12.0% of the total effect, supporting hypothesis H9. Based on these results, the chain mediation model is illustrated in Figures 2, 3.

## 5 Discussion

### 5.1 Sports activities have a significant positive impact on teenagers’ awareness of rules

Sports activities, by providing a structured environment and a clear framework of rules, significantly enhance adolescents’ awareness of rules and their ability to understand social norms. This effect may stem from core elements inherent in sports activities, such as rule adherence, teamwork, and fair competition, which offer opportunities for adolescents to practice and reinforce their awareness of rules.

Furthermore, this study found that the direct effect of sports activities on rule awareness accounts for 40.3% of the total effect, highlighting that sports activities are not only a means of physical exercise but also an important pathway for socialization and the internalization of rules. Through following rules, engaging in teamwork, and experiencing fair competition, adolescents gain practical understanding and internalize the importance of rules, thereby enhancing their rule awareness and understanding of social norms.

Further analysis suggests that the influence of sports activities on rule awareness may operate through multiple mechanisms. First, sports activities typically require participants to follow explicit rules and procedures, and this rule-based constraint helps adolescents develop sensitivity to and awareness of rules. By adhering to specific rules and procedures in sports, adolescents form an increased sensitivity to and commitment to following rules.

Second, the team collaboration and competitive contexts in sports activities provide adolescents with opportunities to practice and internalize rules. Cooperation and competition in team sports notably enhance adolescents’ rule awareness and self-regulation abilities.

Finally, feedback from coaches and peers in sports activities provides a supportive environment for learning rules, further reinforcing the internalization of rule awareness. This social support system contributes to the reinforcement of rule understanding and the internalization process.

### 5.2 The mediating effect of friendship quality on sports activities and adolescents’ awareness of rules

This study finds that friendship quality plays a significant mediating role in the relationship between sports activities and adolescents’ rule awareness. Specifically, teamwork and interaction in sports activities create a natural environment for adolescents to build friendships. Through participating in sports together, adolescents not only experience the joy of teamwork but also learn how to follow rules and respect others through their interactions with peers. High-quality friendships further strengthen adolescents’ understanding of and commitment to rules, thus serving as a bridge between sports activities and rule awareness.

Some scholars argue that both extroverted personality traits and high-quality sports friendships can promote adolescents’ exercise behaviors, with sports friendships serving as a partial mediator in this process (36). Moreover, the impact of friendship quality on adolescents’ rule awareness may be closely related to their social cognitive abilities. Research has shown that the quality of sports friendships is significantly positively correlated with adolescents’ sports participation, self-esteem, and social orientation (37). This suggests that strong friendships may indirectly promote adherence to rules by enhancing adolescents’ self-esteem and social skills.

### 5.3 The mediating effect of emotional intelligence on physical activity and adolescent rule awareness

The mediating effect of emotional intelligence in the relationship between sports activities and adolescents’ rule awareness is a complex and multidimensional research topic, involving fields such as psychology, education, and sociology. In recent years, with the rise of positive psychology and sport psychology, emotional intelligence—recognized as a crucial indicator of emotional regulation and social adaptation—has garnered widespread attention. As an important form of social interaction, sports activities not only promote adolescents’ physical health but may also influence the formation and development of their rule awareness through the mediating role of emotional intelligence.

The mediating role of emotional intelligence between sports activities and rule awareness can be explained from the perspectives of emotional regulation and social cognition. In team sports, adolescents need to perceive the emotional states of both teammates and opponents, understand the impact of emotions on team cooperation, and manage their emotions to maintain harmony and efficiency within the team (38). The enhancement of this emotional regulation ability helps adolescents better understand and adhere to rules in broader social contexts, thereby strengthening their rule awareness.

Furthermore, sports activities provide adolescents with a “laboratory” to practice both emotional intelligence and rule awareness. In these activities, adolescents learn how to balance emotions and rules through observing and imitating the behaviors of coaches and teammates during competition and cooperation. This learning process not only improves their emotional intelligence but also reinforces their understanding and willingness to follow rules.

However, the mediating effect of emotional intelligence in the relationship between sports activities and rule awareness may be influenced by cultural and social environments. Expectations regarding emotional expression and rule adherence vary significantly across cultural contexts. For example, in collectivist cultures, the development of emotional intelligence is often closely related to group harmony, while in individualistic cultures, emotional intelligence may be more associated with personal achievement and self-actualization. Therefore, the moderating role of cultural background should be considered when exploring the mediating effect of emotional intelligence.

Finally, the mediating effect of emotional intelligence may also be realized through neurobiological mechanisms. Recent neuroscience research has shown that emotional regulation and rule adherence involve the collaborative action of brain regions such as the prefrontal cortex, amygdala, and cingulate gyrus (39). Sports activities, by promoting brain plasticity, may enhance the functional connectivity of these brain regions, thereby improving emotional intelligence and rule awareness. Brain imaging studies of adolescent athletes have found that those who engage in sports regularly show significantly higher activation levels in the prefrontal cortex compared to non-athletes, with these activation levels positively correlated with emotional intelligence and rule awareness (40).

### 5.4 The chain-mediated effects of friendship quality and emotional intelligence on physical activity and adolescents’ awareness of rules

The facilitative effect of physical activities on adolescents’ rule consciousness fundamentally arises from synergistic mechanisms bridging social interaction and psychological development. Team-based sports construct cooperative contexts that serve as social theaters for rule enactment, wherein synchronized movements and shared goal attainment not only activate mirror neuron systems to establish neural substrates for empathy (41) but also deepen functional cognition of rules through concrete interactions such as conflict negotiation and role specialization. Within these athletic social fields, high-quality friendships cultivated through sports create unique affective socialization spaces. Continuous emotional feedback and regulatory practices among peers—such as empathetic support during competitive failures or shared emotional resonance in victories—enable adolescents to refine emotion-decoding capacities and adaptive strategies. This dynamic emotional learning process constitutes a micro-level mechanism for emotional intelligence development (42).

As individuals’ emotional intelligence becomes enhanced through social praxis, their rule comprehension transcends superficial behavioral constraints, evolving into internalized capacities for recognizing and responding to socioemotional cues (43). For instance, perceiving anxiety induced by rule violations in collective settings or employing emotional self-regulation to suppress impulsive transgressions. The transformative pathway from behavioral engagement → emotional resonance → cognitive internalization may be amplified within collectivist cultural contexts, where relational self-construal motivates adolescents to associate rule adherence with group belongingness (44). Here, heightened social approval expectations stemming from improved friendship quality catalyze psychological motivations for rule internalization.

Notably, this chained mechanism may exhibit gendered divergence: male adolescents predominantly reinforce functional rule cognition through instrumental reciprocity in athletic teams, whereas females rely more on empathy cultivated within affective bonding to decode rules’ societal significance (45). From a neuroeducation perspective, sports-induced neural reorganization—particularly enhanced connectivity between the prefrontal cortex and limbic system (46)—may provide biological substrates for such socioemotional capacities. Repeated social-motor experiences thus forge synergistic neural networks co-representing emotional regulation and rule cognition.

### 5.5 The promoting role of friendship quality on emotional intelligence and comparison with the reverse pathway

The chained mediation model constructed in this study reveals that physical activity enhances adolescents’ emotional intelligence (EI) by improving friendship quality, which subsequently influences their rule awareness. Additionally, we explore a potential reverse pathway—where EI development may reciprocally foster friendship quality.

Theoretically, high-quality friendships provide individuals with substantial emotional support and interactive experiences, serving as a critical context for emotional socialization. Drawing on social support theory and emotional socialization theory, adolescents in such friendships develop trust, mutual understanding, and affective exchanges. These interactions not only yield positive feedback but also cultivate conflict resolution and emotion regulation skills, thereby enhancing emotional cognition and management capabilities (34). Within the highly interactive context of physical activity, peer support and encouragement are particularly salient. This environment not only elevates friendship quality but also offers a natural platform for practicing and developing EI.

Conversely, scholars have posited that individuals with higher EI may better recognize others’ emotions and communicate effectively, facilitating the establishment and maintenance of high-quality friendships (25). This perspective emphasizes EI as an intrinsic regulatory capacity that may reciprocally strengthen friendship bonds. However, in physical activity settings, adolescents often form friendships rapidly through team collaboration and competitive interactions with immediate feedback. Here, high friendship quality not only provides direct emotional support but also creates external conditions necessary for further EI development. Temporally, physical activity first enhances peer interaction and trust, improving friendship quality, which in turn becomes a prerequisite for EI advancement.

Our analysis suggests that, within physical activity contexts, friendship quality is more likely to act as a “catalyst” for EI development, indirectly promoting rule awareness. While EI may reciprocally influence friendship quality to some extent, existing literature and our data predominantly support the sequential pathway: physical activity initially improves peer relationships, and the positive emotional dynamics within these friendships establish the contextual foundation for emotion regulation and cognitive growth, ultimately driving EI development (47). Future studies should employ longitudinal or experimental designs to investigate dynamic bidirectional effects between EI and friendship quality, thereby comprehensively elucidating their causal interplay.

In summary, this study strengthens the theoretical foundation for the friendship quality → EI mediating pathway, highlighting high-quality peer relationships as a cornerstone of EI enhancement in physical activity contexts. Although the reverse EI → friendship quality pathway is discussed, both empirical and theoretical evidence indicate the former mechanism is more salient in the current framework.

## 6 Conclusion and future outlook

### 6.1 Conclusion

First, there is a significant correlation between sports activities, friendship quality, emotional intelligence, and rule awareness. Second, sports activities can significantly and positively predict adolescents’ rule awareness, making them an important intervention variable for the development of rule awareness in adolescents. Third, friendship quality and emotional intelligence play not only a simple mediating role in the impact of sports activities on adolescents’ rule awareness, but also exert a chain-mediated effect through friendship quality and emotional intelligence. This indirect mediating effect has a profound impact on the development of adolescents’ emotional competencies.

### 6.2 Research advantages and limitations

#### 6.2.1 Advantages

This study pioneers a chain mediation model connecting physical activity, friendship quality, emotional intelligence, and rule consciousness, offering novel insights into their interplay.

#### 6.2.2 Limitations

Findings may lack broader applicability due to a regionally restricted sample (Shandong Province) and reliance on cross-sectional data, necessitating longitudinal research to validate long-term effects.

### 6.3 Future outlook

#### 6.3.1 Theoretical and technological perspectives

Digital sports technologies are driving a paradigm shift in adolescent rule cognition research by elucidating the neuro-social synergistic mechanisms of rule internalization through virtual-reality interactions. Immersive VR/AR environments leverage embodied cognition to reconfigure rule perception, where multisensory simulations enhance the encoding of procedural memory, while wearable devices dynamically decode rule adaptation patterns in sports contexts through multimodal kinematic, physiological, and affective data capture. Concurrently, digital twin platforms map the nonlinear trajectories of cognitive development by tracking social network dynamics. Neuroscientific investigations employing functional near-infrared spectroscopy (fNIRS) reveal prefrontal-limbic co-activation patterns during motor interventions, highlighting neural resonance between cognitive control and emotional regulation. Special education research explores threshold effects of digital technologies in modulating heterogeneous rule cognition among autistic adolescents, whereas cross-cultural neuroimaging studies contrast the neural representations of East Asian li (ritual propriety) and Western legal rationality in rule discipline. Within AI-saturated societies, this field is undergoing a revolutionary transition from behavioral conditioning to endogenous construction. Serving as cognitive scaffolds, digital technologies foster critical rule awareness through blended virtual-physical praxis, enabling organic integration of self-regulated rule generation systems with sociometrical frameworks. This convergence ultimately catalyzes theoretical innovation at the intersection of environmental psychology and cultural neuroscience, advancing our understanding of how technology-mediated experiences reshape the neurobiological and sociocultural substrates of rule cognition.

#### 6.3.2 Practical and operational strategies

To operationalize theoretical insights, this initiative prioritizes five actionable strategies: digital intervention pilots, interdisciplinary curriculum development, intelligent feedback systems, data-driven behavioral analytics, and home-school-community collaboration platforms. The digital intervention pilots involve implementing 8–12 week randomized controlled trials (RCTs) in schools or communities, where wearable devices, dedicated applications, and IoT cloud platforms collectively enable real-time collection of physiological and behavioral data, while AI-powered personalized feedback mechanisms assess impacts on rule awareness, emotion regulation, and team collaboration. Concurrently, interdisciplinary curriculum development integrates expertise from physical education, information technology, psychology, and behavioral science to design AR/VR-enhanced simulated competitive scenarios, supplemented by adaptive mini-games and quizzes on digital platforms to deliver individualized rule cognition training. The intelligent feedback systems employ motion tracking and emotion recognition algorithms to automatically detect rule violations, triggering immediate corrective prompts and reward-based reinforcement, with machine learning iteratively refining feedback protocols based on developmental patterns. Longitudinal behavioral datasets are analyzed through AI modeling to identify at-risk students for tailored interventions, complemented by multi-method evaluations combining surveys, interviews, and observational data to iteratively optimize program efficacy. Finally, the integrated home-school-community platform synchronizes cross-contextual data streams, enabling collaborative monitoring and strategy alignment among parents, teachers, and coaches. Virtual workshops facilitate stakeholder engagement in transitioning adolescents from short-term rule compliance to sustained internalization, thereby establishing an empirical foundation for policy formulation and scalable implementation.

## Data Availability

The raw data supporting the conclusions of this article will be made available by the authors, without undue reservation.
